# 

**Published:** 1904-12-10

**Authors:** 


### NOTICE TO CORRESPONDENTS.

All MSS., Letters, Books for Review, and other matters intended for the Editor should be addressed The Editor, "The Hospital," The Hospital Buildings, 28 & 29 Southampton Street, Strand, W.C.

The Editor cannot undertake to return rejected MS., even when accompanied by stamped directed envelope.

**Notice to Advertisers and Subscribers.**

All Advertisements, Orders for Copies of The Hospital, and Business Communications should be addressed to **THE MANAGER (Not to the Editor),** The Hospital, 28 & 29 Southampton Street, Strand, London, W.C.

The Cost of Subscription, post free, is as follows:—Per week, 3½d.; per quarter, 3s. 3d.; per half-year, 6s. 6d.; per annum, 13s. Foreign and Colonial:—Per annum, 19s. 6d., payable, in all cases, in advance.

**NOTICE.—Vol. XXXVI. of "The Hospital" (April to September 1904), in handsome cloth binding, is now on sale at the office of this Journal, price 10s. 6d. Cases for binding the Numbers contained in this Volume 2s. Index to Vol. XXXVI., 3½d., post free.**

**The Monthly part for December is now ready. Price 1s., by post, 1s. 3d.**

### BOOKS RECEIVED.

A. and C. Black.

"The King's Homeland, Sandringham and North-West Norfolk." By W. A. Dutt.

J. Wright and Co., Bristol.

"Diseases of the Ear." By J. K. Love, M.D.

Baillière, Tindall and Cox.

"Walsham's Handbook of Surgical Pathology." Third edition. Revised and largely rewritten by Herbert J. Paterson, M.B., F.R C S.

"The Present Conditions of Infant Life, and their Effect on the Nation."

"Some Methods of Hypodermic Medication in the Treatment of Inoperable Cancer." By J. A. Shaw-Mackenzie, M.D.

"Manual of Diseases of Women." Ninth Edition. By H. Macnaughton Jones, M.D, M.Ch.

Mather and Crowther, Ltd.

"Practical Advertising, 1904–1905."

The Scientific Press, Limited.

"The Schott Treatment for Chronic Heart Diseases." By R. Greene, F.R.C.P. Third edition.

Consumption Chart, Case Paper, and Pathological Report.

### Medical Appointments.

G. H. S. Blackburne, M.B., B.S.Melb., D.P.H.Camb., Pathologist and Bacteriologist to the Perth Public Hospital, West Australia. Walter Blaxland, F.R.C.S.Eng., Ophthalmic Surgeon to the Perth Public Hospital, West Australia. Frederick C. Carle, M.B.Lond., M.R.C.S., Senior Clinical Assistant, the Throat Hospital, Golden Square, W. Reginald G. Cookson, L.R.C.P.Lond., Government Medical Officer and Vaccinator at Temora, New South Wales. Thomas Arthur Grieves, L.R.C.P., M.R.C.S., Government Medical Officer and Vaccinator at Burraga, New South Wales. Ernest W. Hey Groves, M.D., B.S., B.Sc.Lond., Demonstrator of Anatomy at University College, Bristol. H. A. C. Irving, M.B., Health Officer for the Shire of Lawloit and Public Vaccinator, North-Western District, Victoria. Henry Temple Mursell, M.B., M.C., F.R.C.S.E., Honorary Assistant Surgeon to the Johannesburg Hospital. Ogden Watson Ogden, M.D., B.S.Durh., M.R.C.S.Eng., L.R.C.P.Lond., Honorary Physician to the Hospital for Sick Children, Newcastle-upon-Tyne. P. M. O'Meara, M.B., B.Ch., Health Officer at Coolgardie, West Australia. F. W. L. Pollard, Med. L.Ch.Dub., Medical Officer for the Mellor District of the Blackburn Union. John Pollock, M.B., Health Officer for the Shire of Avon, Victoria. H. P. J. Price, M.R.C.S.Eng., L.S.A., District Medical Officer of the Narberth Union. G. J. Saunders, M.B., Ch.B.Aberd., Certifying Factory Surgeon for the Burghead District, co. Elgin.

St. Thomas's Hospital.—The following House appointments have been made to date from Tuesday, December 6th, 1904:—Resident House Physicians: B. Higham, M.R.C.S., L.R.C.P. (*Extension*); W. Haward, M.B., B.S.Durh., M.R.C.S., L.R.C.P. (*Extension*); A. G. Gibson, B.A., M.B., B.Ch.Oxon., B.Sc.Lond.; K. Takaki, M.R.C.S., L.R.C.P. House Physicians to Out-patients: R. E. Whitting, M.A., M.B., B.C.Cantab.; F. A. Brodribb, M.R.C.S., L.R.C.P. Resident House Surgeons: H. S. Bennett, M.R.C.S., L.R.C.P.; N. C. Carver, B.A., B.C.Cantab., M.R.C.S., L.R.C.P.; A. C. Birt, M.R.C.S., L.R.C.P.; G. T. Birks, M.A., M.B., B.C.Cantab. (*Extension*). House Surgeons to Out-patients: H. A. Kisch, M.R.C.S., L.R.C.P.; G. R. Footner, B.A.Cantab., M.R.C.S., L.R.C.P.; R. E. G. Gray, M.A.Cantab., M.R.C.S., L.R.C.P.; J. C. F. D. Vaughan, M.R.C.S., L.R.C.P. (*Extension*). Obstetric House Physicians: H. I. Pinches, M.A., M.B., B.C.Cantab., M.R.C.S., L.R.C.P. (*Senior*); E. W. Parry, M.R.C.S., L.R.C.P. (*Junior*). Ophthalmic House Surgeon: H. S. Stannus, M.B.Lond., M.R.C.S., L.R.C.P. (*Senior*). A. B. Bradford, M.B., B.S.Durh., M.R.C.S., L.R.C.P. (*Junior*). Special Departments: (Throat) C. N. Sears, M.B., B.S.Lond., M.R.C.S., L.R.C.P.; D. K. Coutts, M.R.C.S., L.R.C.P. (Skin) W. L. Harnett, M.A., M.B., B.C.Cantab., M.R.C.S., L.R.C.P. (*Extension*); F. M. Bulley, B.A.Cantab., M.R.C.S., L.R.C.P. (*Extension*). (Ear) T. Guthrie, M.A., M.B., B.C.Cantab., M.R.C.S., L.R.C.P. (*Extension*); L. E. C. Norbury, M.R.C.S., L.R.C.P.

**"The Hospital" Institutional Library.**

**"Hospitals and Asylums of the World." 4 Vols., with a Portfolio of Plans, complete £12 12s.**

**"Burdett's Hospitals and Charities, 1904." 5s. net.**

**"Burdett's Official Nursing Directory." 8s. net.**

**"Cottage Hospitals, General, Fever, and Convalescent." 10s. 6d.**

**"Uniform System of Accounts for Hospitals and Public Institutions." New Edition. 4s. net.**

**"Hospital Expenditure: The Commissariat." 2s. 6d.**

**"A Legal Handbook for the Use of Hospital Authorities." 2s. 6d.**

**"The Cottage Hospital Case Book or Register of Patients." 10s. and 12s. 6d.**

**All these are published by the Scientific Press, Ltd., and may be obtained through any bookseller, or direct from the publishers, 28 and 29 Southampton Street, Strand, London, W.C.**

### Xmas Presents for Nurses.

### For Probationer Nurses.

**THE NURSING PROFESSION: How and Where to Train.**
By Sir Henry Burdett, K.C.B.
Bound in Cloth, 2/- net, 2/4 post free.

**NURSING: ITS THEORY AND PRACTICE.**
A Complete Text-book of Medical, Surgical and Monthly Nursing.
By Percy G. Lewis, M.D.
Bound in cloth, 3/6 post free.

### For Post Graduate Nurses.

**A HANDBOOK FOR NURSES.**
By J. K. Watson, M.D.
Profusely Illustrated. Bound in cloth.
5/- net, 5/4 post free.

**NURSING: ITS PRINCIPLES AND PRACTICE.**
By Isabel Adams Hampton.
New Edition, cloth.
7/6 net, 7/10 post free.

### For Nurses of all Grades.

**THE NURSES' PRONOUNCING DICTIONARY OF MEDICAL TERMS AND NURSING TREATMENT.**
By Honnor Morten.
Fifth Edition, Edited and Revised with phonetic pronunciation,
By Mary I. Burdett.
Suitable size for apron pocket.
Cloth, 2/- post free.
Handsome leather gilt, 2/6 net, 2/8 post free.

### For Surgical Nurses.

**SURGICAL WARD-WORK AND NURSING.**
By Alexander Miles, M.D.
Second edition, containing illustrations and particulars of the instruments used in various operations.
Cloth, 3/6 net, 3/10 post free.

**PRACTICAL GUIDE TO SURGICAL BANDAGING AND DRESSINGS.**
By Wm. Johnson Smith, F.R.C.S.
Suitable size for apron pocket.
Cloth, 2/- post free.

### For Hospital Sisters.

**HOSPITAL SISTERS AND THEIR DUTIES.**
By Eva C. E. Lückes,
Matron London Hospital.
Third Edition.
Cloth, 2/6 post free.

**SYLLABUS OF LECTURES TO NURSES.**
By Andrew Davidson, M.D.
Cloth, 1/- post free.

SEND POSTCARD FOR NEW CATALOGUE OF NURSING MANUALS.

**THE SCIENTIFIC PRESS, LTD.**
*Publishers of Nursing Text-Books, Case-Books, Charts, &c.,*
**28 & 29 Southampton St., Strand, London, W.C.**

